# Prevalence and Associated Factors of Bullying Experienced by Community Patients With Serious Mental Disorders: Cross-Sectional Study

**DOI:** 10.2196/75027

**Published:** 2025-09-03

**Authors:** Yazhuo Qi, Meiqi Wang, Zhen Wei, Wenyu Wang, Kaixian Wang, Long Sun

**Affiliations:** 1 Centre for Health Management and Policy Research, School of Public Health, Cheeloo College of Medicine, Shandong University Jinan, Shandong Province China

**Keywords:** serious mental disorders, bullying, stigma, sleep quality, family functioning

## Abstract

**Background:**

Individuals with serious mental disorders (SMDs) are frequently exposed to bullying, which can severely affect their well-being. However, research on this issue remains limited in the Chinese context.

**Objective:**

This study aimed to examine the prevalence and associated factors of bullying experienced by individuals with SMDs in China, with the goal of providing evidence that may inform strategies to improve their quality of life.

**Methods:**

A multistage stratified random sampling method was used to survey 486 community patients with SMDs in Shandong province, China. Participants self-reported experiences of bullying. The Stigma Scale for Chronic Illnesses, 8-item version was used to measure self-stigma; the Pittsburgh Sleep Quality Index assessed sleep quality; and the Family Apgar Scale evaluated family functioning. Single-factor analysis, logistic regression, and negative binomial regression were used to analyze the associations between variables.

**Results:**

The prevalence rate of bullying experienced by individuals with SMDs was 42.4% (206/486). Higher levels of self-stigma were significantly associated with increased odds of being bullied (odds ratio [OR] 1.041, 95% CI 1.017-1.065; *P*<.001), while better family functioning was associated with lower odds of experiencing bullying (OR 0.913, 95% CI 0.850-0.980; *P*=.01). Verbal abuse was associated with poorer sleep quality (OR 1.056, 95% CI 1.004-1.111; *P*=.04), particularly when reported as perpetrated by caregivers (OR 1.068, 95% CI 1.029-1.108; *P*<.001). In addition, age (*P*<.001), education level (*P*=.02), only-child status (*P*=.04), and diagnosis type (*P*=.03) were significantly related to bullying experiences.

**Conclusions:**

Higher levels of self-stigma were associated with experiencing bullying among individuals with SMDs, whereas better family functioning was associated with lower odds of being bullied. Verbal abuse—especially by caregivers—was associated with poorer sleep quality. These findings suggest that reducing self-stigma and strengthening family support may help improve quality of life for individuals with SMDs.

## Introduction

### Background

Serious mental disorders (SMDs) are mental disorders in which the symptoms are so severe that an individual’s social functioning is significantly impaired, preventing them from accurately understanding their health status or objective reality or managing their own affairs [1]. In China, the spectrum of SMDs is diverse and includes conditions such as schizophrenia, paranoid mental disorder, bipolar disorder, schizoaffective disorder, epileptic mental disorder, and intellectual disability associated with mental disorders [2]. Official figures show that approximately 4.7% of people in China experience SMDs, highlighting the significant prevalence of these conditions [2]. The impact of mental disorders on public health is profound. In 2021, they ranked among the leading causes of disease burden, accounting for 1554.2 disability-adjusted life years per 100,000 people globally [3]. This metric underscores the extent to which mental disorders contribute to the overall loss of health and productivity in the population. Therefore, mental disorders are an important public health issue that requires more attention and research [4].

Patients with SMDs often face multiple challenges and societal discrimination, and bullying is one of the most serious problems [5]. Discrimination refers to unfair treatment based on group characteristics, whereas bullying is repeated, intentional harm or intimidation targeting an individual, often involving a power imbalance. People with SMDs are often misunderstood as being violent—in reality, they are more likely to experience violence and bullying rather than commit such acts [6]—and this widespread misconception contributes to the self-stigma and discrimination faced by those with mental health conditions, further exacerbating their vulnerabilities. Studies in the United States have shown that experiencing bullying can affect the course of illness and treatment outcomes for patients with SMDs [7] and can also reduce their quality of life [8]. An Australian study demonstrated a longitudinal association between being bullied and anxiety and depressive disorders, showing that people with SMDs who are bullied often develop severe anxiety and depressive symptoms, which leads to a further deterioration in their mental health status [9]. In addition, a study conducted in Canada, Finland, Germany, and Sweden found that exposure to bullying, depression, and medication nonadherence were all associated with poorer real-life functioning in men with schizophrenia” (assuming that “exposure to bullying” can substitute for “victimization” in the cited work) [10]. To reduce the negative impact of bullying on patients with SMDs, all sectors of society need to work together to increase public awareness of the issue, strengthen mental health support and legal protection, and develop and implement effective antibullying measures. These efforts are crucial for improving the living conditions and overall well-being of people with SMDs.

Previous studies have reported prevalence estimates and risk factors for bullying experienced by individuals with SMDs. In the largest study of people with a major mental illness in the United States, 44% reported experiencing violence [8]. There are many factors influencing exposure to bullying in patients with SMDs. A Serbian study of clinical patients with schizophrenia found that sex was a key factor, with men being more likely to experience physical bullying [11]. A longitudinal study in the United Kingdom confirmed that younger age, single marital status, homelessness, and limited contact with family were associated with a significantly increased risk of being bullied [5]. In addition, studies in the United States [8] and Egypt [12] have shown that patients with SMDs are mainly bullied by acquaintances, followed by family members [13]. However, most of the studies on bullying have been conducted mainly in Europe [5,11], the United States [8], and Australia [14]. No survey has examined bullying experienced by community patients with SMDs in China. This gap indicates that research on bullying in this population in China remains limited. Understanding the prevalence and nature of bullying experienced by patients with SMDs in China is crucial for developing effective interventions tailored to the country’s specific cultural and social context.

### Objectives

Due to the lack of relevant research, the specific experiences or related factors of bullying experienced by Chinese patients with SMDs remain unclear. Given China’s unique cultural and social context, these experiences may differ significantly from those reported in Western countries [15]. Cultural factors such as strong family ties, collectivist values, self-stigma surrounding mental illness, and differences in mental health care accessibility may influence both the prevalence and nature of bullying experienced by patients with SMDs in China [16]. Moreover, social perceptions of mental illness and institutional factors may shape how patients with SMDs navigate their daily lives, potentially increasing their vulnerability to bullying [17].

Therefore, this study aimed to report the prevalence of bullying experienced by community patients with SMDs in China and to examine the associations among disease-specific factors, sleep quality, self-stigma, and family functioning that influence exposure to bullying among patients with SMDs, while controlling for sociodemographic variables. This comprehensive approach can help us understand and address the bullying issues faced by people with SMDs, ultimately improving their quality of life and enhancing the recovery process. In addition, the findings could provide an important scientific basis for developing more effective prevention and intervention strategies to promote the health and well-being of people with SMDs on a broader societal level.

## Methods

### Study Design and Sampling

This cross-sectional study investigated individuals with SMDs residing in Shandong province in eastern China. A multistage stratified random sampling method was used to ensure representativeness and reliability [18]. First, the 16 major cities in Shandong province were categorized into 2 groups based on their respective gross domestic product. One city was then randomly chosen from each group, resulting in the selection of Jinan and Zaozhuang. Within these 2 cities, 1 administrative district was randomly chosen, specifically Zhangqiu and Taierzhuang, respectively. Finally, 6 townships and communities were randomly selected as survey sites within each administrative district. Participants were recruited from the National Information System for Severe Mental Disorders, which records all patients receiving long-term community-based management [19]. Local community health institutions assisted with recruitment by providing patient lists and contacting eligible individuals or their guardians to explain the study and confirm voluntary participation [20]. Face-to-face interviews were arranged at participants’ homes or local health centers after obtaining written informed consent [21]. Inclusion criteria were registration in the National Information System for Severe Mental Disorders, residence in the community for at least 6 months, and ability to communicate independently or with caregiver support. A total of 486 individuals with SMDs were enrolled.

### Data Collection

The study was carried out from August to September 2022. Before administering the survey, all investigators—graduate students proficient in both the research topic and questionnaire design—underwent professional training. Data were collected through 1-on-1, on-site interviews using a predesigned questionnaire [22,23]. Recognizing the unique circumstances of the respondents, the investigators sought the assistance of the patient’s caregiver during the interviews. To ensure familiarity with the patient’s daily experiences, only caregivers who had lived with and provided care for the patient for at least 6 months were eligible to respond [24]. In cases where respondents faced cognitive impairment or difficulty answering, their caregiver provided responses on their behalf [25]. The investigators were trained to use standardized definitions and provide clear examples of key terms such as “bullying” and “verbal abuse” to minimize subjective variation in interpretation [26]. Interviews were conducted either at participants’ homes or local health centers, based on their convenience and preference, and responses were recorded directly onto the questionnaire. After the survey, at least 2 trained students meticulously reviewed each questionnaire to ensure its validity, double checking and completing missing or unclear data entries as necessary [27,28].

### Ethical Considerations

This study was approved by the institutional review board of the Shandong University School of Public Health (LL20210803). All participants volunteered and provided written informed consent. For those who were illiterate or semiliterate, written informed consent was obtained from their legal guardian [29]. All collected information was anonymized using unique ID codes. In accordance with the institutional review board guidelines, no financial compensation was provided to participants to avoid potential coercion. The manuscript contains no personally identifiable information, and no images requiring additional consent were used. The research strictly adhered to the principles of the Declaration of Helsinki and Chinese ethical regulations governing human participant research.

### Measures

#### Bullying Experiences

Bullying experiences in this study were assessed via self-report using 2 primary questions: “Have you ever been verbally abused by a caregiver or other person?” “Have you ever been physically assaulted by a caregiver or other person?” Respondents were required to answer with either “yes” or “no.” Those who indicated that they had not encountered bullying were assigned a score of “0,” whereas respondents who reported experiencing bullying were assigned a score of “1.” Subsequently, interviewers delved deeper to ascertain the specific types of bullying encountered by asking, “Have you experienced any of the following behaviors by caregivers/others?” Response options included “discrimination,” “verbal abuse,” “beatings,” “imprisonment,” “cold starvation,” “forced physical labor,” “bondage,” “other,” and “none.” All respondents who answered “no” to experiences of bullying were designated as the control group, labeled as “with no bullying,” and those who reported any form of bullying were categorized under “any bullying.” The interviewers then calculated bullying scores by summing the reported instances of various types of bullying for each respondent, categorizing them as either “verbal bullying” or “physical bullying.”

#### Sociodemographic Variables

The primary sociodemographic variables examined in this study included sex, age, education level, sibling status, and marital status. These variables were coded numerically to facilitate analysis. Sex was coded as male (1) and female (2). Age categories, derived from participants’ date of birth, were coded as <45 years (1), 45 to 64 years (2), and >64 years (3). Educational level was coded as illiteracy or semiliteracy (1), primary school (2), and middle school or above (3). Sibling status was coded as single child (1) or other (2). Marital status was simplified and coded as married (1) and other (2), with the latter category encompassing single, divorced, widowed, and other statuses due to their small proportions.

#### Illness Information

SMDs include 6 distinct types: schizophrenia, schizoaffective disorder, paranoid psychosis, bipolar disorder, mental disorders resulting from epilepsy, and intellectual disability associated with mental disorders accompanied by mental disorders [30]. However, due to the relatively small proportion of patients with the latter 5 types, This study grouped SMD types into schizophrenia (1) and other (2) to assess patients’ disease profiles.

To assess patients’ current psychiatric symptoms, we used the 18-item Brief Psychiatric Rating Scale, a widely recognized and validated tool for quantifying the severity of mental disorders [31]. Illness duration was measured in years (ie, duration of SMD), based on self-reports from patients or their caregivers. In addition, we used the Stigma Scale for Chronic Illnesses, 8-item version (SSCI-8) to evaluate patients’ self-stigma toward mental illness [32].

#### Sleep Quality

Sleep quality was evaluated using the Pittsburgh Sleep Quality Index [33]. This comprehensive scale comprises 7 dimensions aimed at assessing individual sleep quality and the severity of sleep disturbances. These dimensions include subjective sleep quality, sleep duration, sleep disturbance, sleep latency, habitual sleep efficiency, daytime dysfunction, and sleep medication use. Each dimension is scored from 0 to 3, yielding a total score ranging from 0 to 21. Higher scores indicate poorer sleep quality. The scale assesses respondents’ experiences over the past month. It is widely used in studies evaluating the sleep quality of the Chinese population due to its high reliability and validity [34].

#### Family Functioning

This study used the Family Apgar Scale to evaluate family functioning [35]. Renowned for its brevity and clarity, this scale has found widespread application in assessing family dynamics [36]. In China, notably, it has demonstrated efficacy as a diagnostic tool for identifying individuals with family dysfunction [37]. The scale comprises 5 items measuring adaptation, partnership, growth, affection, and closeness. Scores from the 5 items are summed to yield a total score ranging from 0 to 10, with 0 to 3 indicating severe dysfunction, 4 to 6 moderate dysfunction, and 7 to 10 good functioning [35]. In this study, the Cronbach α coefficient for the scale was 0.934, indicating robust reliability.

### Data Analyses

The primary analysis was conducted using SPSS software (version 27.0; IBM Corp). Continuous variables were presented as means and SDs, while categorical data were reported as frequencies and percentages. To assess the mean differences between the “any bullying” and “with no bullying” groups, either a chi-square test or a 1-way ANOVA was used. As the outcome was binary (yes or no), we used logistic regression to assess associations between the variables and experiences of bullying, reporting odds ratios (ORs) with 95% CIs as measures of association rather than causality. For the multivariable logistic regression analysis, dummy variables were established for multicategory variables to explore factors associated with experiences of bullying [38].

In addition, generalized estimating equation (GEE) analyses were used to investigate factors linked to the number of types of bullying behaviors experienced. Given that the dependent variable constituted count data, we opted for a negative binomial distribution to model it [39]. Within the GEE framework, we conducted Wald tests for hypothesis testing to determine whether the effect of independent variables on the number of types of bullying behaviors experienced was significant. Throughout the analysis, we controlled for variables such as age and sex to mitigate the influence of confounding factors [40]. It is crucial to note that all hypothesis tests in this study were 2-tailed, with a significance level set at .05 [41]. Therefore, results were deemed statistically significant when the *P* value was <.05.

## Results

### Sample Characteristics and Single-Factor Analysis

This study enrolled 486 community patients diagnosed with SMDs, among whom 206 (42.4%) reported experiencing bullying by caregivers or others. Of these 206 participants, 169 (82%) had been verbally abused, and 113 (54.9%) had been physically assaulted. Moreover, of those who experienced bullying, 49.5% (102/206) were male, and 50.5% (104/206) were female. In terms of age, 40.8% (84/206) were aged <45 years, 38.3% (79/206) were aged between 45 and 64 years, and 20.9% (43/206) were aged >64 years. Regarding educational attainment, 32% (66/206) were illiterate or semiliterate, 22.8% (47/206) had a primary education, and 45.1% (93/206) had a middle school or above. This reflects a relatively low level of education among patients with SMDs, particularly in the less developed regions of China, where mental health literacy and access to psychiatric services remain limited.

The vast majority (190/206, 92.2%) of the participants were non–only children. This is consistent with the demographic profile of individuals born before or during the early stages of China’s 1-child policy and may also reflect traditional extended family structures in rural Chinese settings. The mean score on the SSCI-8 was 22.31 (SD 8.94).

Of the 169 participants who experienced verbal abuse from caregivers or others, 82 (48.5%) were male, and 87 (51.5%) were female; in terms of age, 75 (44.4%) participants were aged <45 years, 65 (38.5%) were aged between 45 and 64 years, and 29 (17.1%) were aged >64 years. Educational backgrounds varied, with 33.7% (57/169) being illiterate or semiliterate, 22.5% (38/169) having a primary education, and 43.8% (74/169) having a middle school or above. The majority (158/169, 93.5%) were non–only children. The mean score on the SSCI-8 was 22.60 (SD 8.91).

Of the 113 participants who experienced physical assault by caregivers or others, 53 (46.9%) were aged <45 years, 43 (38.1%) were aged between 45 and 64 years, and 17 (15%) were aged >64 years. Regarding educational levels, 30.1% (34/113) were illiterate or semiliterate, 23% (26/113) had a primary school education, and 46.9% (53/113) had a middle school or above. The majority (103/113, 91.2%) were non–only children. The mean score on the SSCI-8 was 22.24 (SD 8.74).

As shown in Table 1, the variables related to experiencing bullying included sex (*χ*²_1_=5.8; *P*=.02), age (*χ*²_2_=13.0; *P*=.001), education (*χ*²_2_=8.9; *P*=.01), sibling status (*χ*²_1_=6.1; *P*=.01), and self-stigma (*F*_2,483_=2.489; *P*<.001). These strata reflect social structure differences: middle-aged adults may face more isolation, only children lack sibling support, and education levels affect coping ability. Traditional Chinese family roles may also shape sibling-related caregiving dynamics. The variables associated with experiencing verbal abuse were sex (*χ*²_1_=4.3; *P*=.04), age (*χ*²_2_=18.7; *P*<.001), education (*χ*²_2_=7.1; *P*=.03), and self-stigma (*F*_2,483_=2.735; *P*<.001). The variables associated with experiencing physical assault were age (*χ*²_2_=18.9; *P*<.001), education (*χ*²_2_=7.2; *P*=.03), sibling status (*χ*²_1_=6.6; *P*=.01), and self-stigma (*F*_2,483_=1.878; *P*=.004). Additional information is presented in Table 1.

**Table 1:** Demographic and clinical characteristics of community patients with serious mental disorders (SMDs) by bullying exposure status (N=486).

Variables		Experiences of bullying					*F* test (*F*_*df*_) or chi-square (*χ*²_*df*_)					
		With no bullying (n=280)	Verbal bullying (n=169)	Physical bullying (n=113)	Any bullying (n=206)	Verbal bullying vs with no bullying			Physical bullying vs with no bullying		Any bullying vs with no bullying	
**Sex, n (%)**								*χ*²_1_=4.3^a^		*χ*²_1_=1.8		*χ*²_1_=5.8^a^
	Male	108 (38.6)	82 (48.5)	52 (46)	102 (49.5)							
	Female	172 (61.4)	87 (51.5)	61 (54.0)	104 (50.5)							
**Age groups (y), n (%)**								*χ*²_2_=18.7^b^		*χ*²_2_=18.9^b^		*χ*²_2_=13.0^c^
	<45	72 (25.7)	75 (44.4)	53 (46.9)	84 (40.8)							
	45-64	125 (44.6)	65 (38.5)	43 (38.1)	79 (38.3)							
	>64	83 (29.6)	29 (17.1)	17 (15)	43 (20.9)							
**Educational levels, n (%)**								*χ*²_2_=7.1^a^		*χ*²_2_=7.2^a^		*χ*²_2_=8.9^a^
	Illiterate or semiliterate	97 (34.6)	57 (33.7)	34 (30.1)	66 (32)							
	Primary school	91 (32.5)	38 (22.5)	26 (23)	47 (22.8)							
	Middle school or above	92 (32.9)	74 (43.8)	53 (46.9)	93 (45.1)							
**Sibling status, n (%)**								*χ*²_1_=3.5		*χ*²_1_=6.6^a^		*χ*²_1_=6.1^a^
	Single child	8 (2.9)	11 (6.5)	10 (8.8)	16 (7.8)							
	Other	272 (97.1)	158 (93.5)	103 (91.2)	190 (92.2)							
**Marital status, n (%)**								*χ*²_1_=1.0		*χ*²_1_=0.9		*χ*²_1_=0.4
	Married	192 (68.6)	108 (63.9)	72 (63.7)	136 (66)							
	Other	88 (31.4)	61 (36.1)	41 (36.3)	70 (34)							
**Types of SMDs, n (%)**								*χ*²_1_=1.4		*χ*²_1_=1.1		*χ*²_1_=2.3
	Schizophrenia	170 (60.7)	112 (66.3)	75 (66.4)	139 (67.5)							
	Other	110 (39.3)	57 (33.7)	38 (33.6)	67 (32.5)							
BPRS^d^ score, mean (SD)		52.93 (20.14)	58.91 (21.87)	57.27 (21.77)	58.92 (22.14)	*F*_2,483_=0.966			*F*_2,483_=0.835		*F*_2,483_=0.978	
Duration of SMD (y), mean (SD)		23.33 (13.61)	23.15 (13.30)	23.75 (12.47)	23.52 (13.16)	*F*_2,483_=1.031			*F*_2,483_=0.895		*F*_2,483_=1.071	
Self-stigma: SSCI-8^e^ score, mean (SD)		17.82 (9.29)	22.60 (8.91)	22.24 (8.74)	22.31 (8.94)	*F*_2,483_=2.735^b^			*F*_2,483_=1.878^c^		*F*_2,483_=2.489^b^	
Poor sleep quality: PSQI^f^ score, mean (SD)		8.08 (4.26)	8.89 (4.22)	8.60 (4.22)	8.86 (4.22)	*F*_2,483_=1.473			*F*_2,483_=1.446		*F*_2,483_=1.684^a^	
Family functioning: FAS^g^ score, mean (SD)		7.23 (3.16)	6.36 (3.27)	5.99 (3.46)	6.44 (3.34)	*F*_2,483_=1.519			*F*_2,483_=1.712		*F*_2,483_=1.273	

^a^*P*<.05.

^b^*P*<.001.

^c^*P*<.01.

^d^BPRS: Brief Psychiatric Rating Scale.

^e^SSCI-8: Stigma Scale for Chronic Illnesses, 8-item version.

^f^PSQI: Pittsburgh Sleep Quality Index.

^g^FAS: Family Apgar Scale.

### Logistic Regression Analysis of the Factors Influencing Different Types of Bullying Experiences

Logistic regression models were used to analyze factors related to bullying experiences. The results of the 3 logistic regression models are presented in Table 2.

**Table 2:** Multivariable logistic regression analysis of factors associated with distinct subtypes of bullying experiences (verbal, physical, or any bullying) among community patients with serious mental disorders (SMDs; N=486).

Variables		Any bullying experience (n=206) vs with no bullying experience (n=280), OR^a^ (95% CI)	Verbal bullying experience (n=169) vs with no bullying experience (n=280), OR (95% CI)	Physical bullying experience (n=113) vs with no bullying experience (n=280), OR (95% CI)
Sex: male (reference: female)		1.483 (0.951-2.314)	1.354 (0.838-2.189)	1.108 (0.631-1.947)
**Age groups (y; reference <45)**				
	45-64	2.795 (1.528-5.112)^b^	3.619 (1.877-6.975)^b^	4.877 (2.212-10.753)^b^
	>64	1.400 (0.823-2.381)	1.756 (0.969-3.182)	2.062 (0.996-4.269)
**Educational levels (reference: illiterate)**				
	Primary school	0.796 (0.483-1.311)	0.912 (0.534-1.558)	0.675 (0.367-1.238)
	Middle school or above	0.566 (0.346-0.925)^c^	0.596 (0.350-1.013)	0.576 (0.315-1.052)
Sibling status: single child (reference: other)		2.894 (1.080-7.760)^c^	2.449 (0.844-7.105)	3.278 (1.064-10.099)^c^
Marital status: married (reference: other)		1.369 (0.843-2.224)	1.176 (0.705-1.961)	1.203 (0.666-2.173)
Type of SMD: schizophrenia (reference: other)		1.598 (1.055-2.420)^c^	1.497 (0.964-2.325)	1.584 (0.952-2.635)
BPRS^d^ score		1.001 (0.991-1.012)	1.001 (0.990-1.103)	0.996 (0.983-1.010)
Duration of SMD (y)		1.011 (0.995-1.028)	1.009 (0.992-1.027)	1.020 (0.999-1.041)
Self-stigma		1.041 (1.017-1.065)^b^	1.046 (1.020-1.072)^b^	1.043 (1.014-1.073)^e^
Poor sleep quality		1.056 (1.004-1.111)^c^	1.061 (1.005-1.120)^c^	1.053 (0.988-1.122)
Family functioning		0.953 (0.897-1.012)	0.946 (0.888-1.009)	0.913 (0.850-0.980)^c^

^a^OR: odds ratio.

^b^*P*<.001.

^c^*P*<.05.

^d^BPRS: Brief Psychiatric Rating Scale.

^e^*P*<.01.

After controlling for relevant sociodemographic variables, we developed 3 models. In model 1, bullying experiences were used as the dependent variable. The results indicated that age group (OR 2.795, 95% CI 1.528-5.112; *P*<.001), education level of middle school or above (OR 0.566, 95% CI 0.346-0.925; *P*=.02), being an only child (OR 2.894, 95% CI 1.080-7.760; *P*=.04), having schizophrenia (OR 1.598, 95% CI 1.055-2.420; *P*=.03), and self-stigma (OR 1.041, 95% CI 1.017-1.065; *P*<.001) were associated with increased bullying experiences. Conversely, sleep quality (OR 1.056, 95% CI 1.004-1.111; *P*=.04) showed a negative association with bullying experiences.

In model 2, verbal abuse experiences were used as the dependent variable. The results showed that age group (OR 3.619, 95% CI 1.877-6.975; *P*<.001) and self-stigma (OR 1.046, 95% CI 1.020-1.072; *P*<.001) were associated with increased verbal abuse experiences. These findings may reflect age-related sensitivity to self-stigma and communication challenges within caregiving relationships. In Chinese society, where self-stigma toward mental illness remains pervasive, such attitudes may contribute to verbal abuse by caregivers or others. By contrast, sleep quality (OR 1.061, 95% CI 1.005-1.120; *P*=.03) was negatively correlated with experiences of verbal abuse.

In model 3, physical assault experiences were used as the dependent variable. The results revealed that age group (OR 4.877, 95% CI 2.212-10.753; *P*<.001), single-child status (OR 3.278, 95% CI 1.064-10.099; *P*=.04), self-stigma (OR 1.043, 95% CI 1.014-1.073; *P*=.003), and lower family functioning (OR 0.913, 95% CI 0.850-0.980; *P*=.01) were associated with increased experiences of physical assault. In the Chinese cultural context, family functioning plays a central role in caregiving for people with mental illness because institutional support remains limited, especially in rural areas. A dysfunctional family environment may increase the likelihood of abusive behaviors by caregivers or others.

### GEE Analysis of the Factors Influencing the Number of Types of Bullying Experiences Across Different Demographics

We used the GEE method to examine the factors influencing the frequency of bullying experiences across various types. Table 3 presents the outcomes of the GEE model.

**Table 3:** Generalized estimating equation models examining the predictors of bullying frequency by perpetrator type (caregivers and others) among community patients with serious mental disorders (SMDs; N=486).

Variables		Any bullying experience (n=206) vs with no bullying experience (n=280), OR^a^ (95% CI)	Any bullying experience by caregivers (n=153) vs with no bullying experience (n=280), OR (95% CI)	Any bullying experience by others (n=143) vs with no bullying experience (n=280), OR (95% CI)
Sex: male (reference: female)		1.143 (0.816-1.602)	1.063 (0.740-1.526)	1.042 (0.720-1.508)
**Age groups (y; reference: <45)**				
	45-64	0.607 (0.438-0.840)^b^	0.566 (0.400-0.800)^b^	0.596 (0.428-0.831)^b^
	>64	0.402 (0.276-0.585)^c^	0.361 (0.239-0.545)^c^	0.411 (0.248-0.681)^c^
**Education levels (reference: illiterate)**				
	Primary school	0.765 (0.523-1.117)	0.694 (0.465-1.036)	1.065 (0.653-1.738)
	Middle school or above	1.059 (0.726-1.544)	1.138 (0.759-1.704)	1.199 (0.784-1.833)
Sibling status: single child (reference: other)		1.965 (1.255-3.078)^b^	1.918 (1.142-3.220)^d^	1.111 (0.680-1.815)
Marital status: married (reference: other)		1.221 (0.893-1.670)	1.183 (0.848-1.651)	0.999 (0.716-1.395)
Type of SMD: schizophrenia (reference: other)		1.330 (0.977-1.812)	1.323 (0.951-1.838)	1.439 (1.037-1.997)^d^
BPRS^e^		0.996 (0.987-1.005)	0.996 (0.987-1.005)	0.999 (0.990-1.008)
Duration of SMD (y)		1.012 (1.000-1.024)	1.012 (0.999-1.026)	1.013 (1.002-1.025)^d^
Self-stigma		1.030 (1.012-1.048)^c^	1.032 (1.014-1.050)^c^	1.037 (1.017-1.057)^c^
Poor sleep quality		1.068 (1.029-1.108)^c^	1.068 (1.026- 1.112)^b^	1.023 (0.983-1.064)
Family functioning		0.945 (0.912-0.979)^b^	0.942 (0.906-0.980)^b^	0.965 (0.925-1.0064)

^a^OR: odds ratio.

^b^*P*<.01.

^c^*P*<.001.

^d^*P*<.05.

^e^BPRS: Brief Psychiatric Rating Scale.

Model 1 revealed that age groups (45-64 y: OR 0.607, 95% CI 0.438-0.840; *P*=.003; >64 y: OR 0.402, 95% CI 0.276-0.585; *P*<.001), single-child status (OR 1.965, 95% CI 1.255-3.078; *P*=.003), self-stigma (OR 1.030, 95% CI 1.012-1.048; *P*<.001), poor sleep quality (OR 1.068, 95% CI 1.029-1.108; *P*<.001), and better family functioning (OR 0.945, 95% CI 0.912-0.979; *P*=.002) as associated with lower odds of experiencing bullying by any perpetrator. The higher vulnerability of only children to repeated bullying may be associated with the caregiving burden concentrated on a single family member in the context of China’s 1-child policy, which limits sibling support and often places intensified pressure on both caregivers and patients.

Model 2 used the number of types of bullying by caregivers as a predictor, with findings indicating that age group (45-64 y: OR 0.566, 95% CI 0.400-0.800; *P*=.001; >64 y: OR 0.361, 95% CI 0.239-0.545; *P*<.001), single-child status (OR 1.918, 95% CI 1.142-3.220; *P*=.01), self-stigma (OR 1.032, 95% CI 1.014-1.050; *P*<.001), poor sleep quality (OR 1.068, 95% CI 1.026-1.112; *P*=.001), and lower family functioning (OR 0.942, 95% CI 0.906-0.980; *P*=.003) was associated with higher odds of caregiver-related bullying. This suggests that in the Chinese sociocultural context, where families are often the sole caregivers for individuals with SMDs, weakened family functioning can be associated with higher odds of emotional or physical abuse from caregivers.

Model 3 used the number of types of bullying by other people as a predictor. The results demonstrated that age groups (45-64 y: OR 0.596, 95% CI 0.428-0.831; *P*=.002; >64 y: OR 0.411, 95% CI 0.248-0.681; *P*<.001), type of SMD (OR 1.439, 95% CI 1.037-1.997; *P*=.03), duration of SMD (years; OR 1.013, 95% CI 1.002-1.025; *P*=.03), and self-stigma (OR 1.037, 95% CI 1.017-1.057; *P*<.001) significantly influenced the likelihood of being bullied by others. The association between longer illness duration and external bullying may reflect persistent social exclusion and labeling common in Chinese communities, where mental illness is still widely stigmatized.

## Discussion

### Principal Findings

This study found a bullying prevalence rate of 42.4% (206/486) among community patients with SMDs. Self-stigma emerged as a significant factor associated with bullying experienced by individuals with SMDs, whereas family functioning seemed to play a potentially protective role. Concerning sleep quality, verbal abuse was associated with poorer sleep for individuals with SMDs, whereas physical abuse showed no such association. Notably, bullying by caregivers was more strongly associated with poorer sleep quality. Furthermore, sociodemographic factors such as age, education, only-child status, and type of SMD also showed significant associations with bullying experiences among individuals with SMDs.

The prevalence rate found in this study is lower than the rates reported in previous studies conducted in the United States (82%), Sweden (67%), and low-income rural areas of Ethiopia (60.7%) [13,42,43]. Such differences may be related to variations in social support systems, access to health care, and cultural attitudes toward mental illness. Furthermore, data from the Global Burden of Disease Study 2019 show a steady increase from 1990 to 2019 in the burden of anxiety and depression associated with exposure to bullying, with the largest increases in disability-adjusted life years observed in high-income regions [44]. These findings suggest that bullying is a widespread and persistent mental health issue globally, highlighting the need for context-specific interventions and mental health policies that address both structural vulnerabilities and social stigma.

Stigma related to mental illness includes public stigma, which involves negative stereotypes and societal discrimination, and self-stigma, in which individuals internalize these negative beliefs [16]. This study found that higher self-stigma levels were significantly associated with bullying experiences, such as verbal abuse and physical assault. Although the cross-sectional design limits causal conclusions, the literature suggests that self-stigma may both result from and be associated with increased vulnerability to abuse [45]. Self-stigma may lead patients to avoid disclosing their condition or seeking help [46], which can contribute to misunderstandings and social exclusion [47]. Public stereotypes portraying people with SMDs as dangerous or unreliable [48], combined with stigma and discrimination [49], may be related to bullying [50]. Moreover, self-stigma is associated with lower patient self-esteem and self-efficacy [51], which may lead individuals with SMDs to become more passive and endure abuse silently, potentially perpetuating the cycle of mistreatment [52]. Our findings add to the literature by showing this association between self-stigma and bullying in a Chinese community sample with SMDs. Given the strong role of family and collectivist culture in Chinese society, individuals with SMDs may experience greater internalized shame and pressure to conceal their illness, which could be related to being bullied. This suggests the importance of addressing self-stigma as part of efforts to reduce bullying and improve patient well-being.

This study adds new insight to existing literature by distinguishing the differing associations of verbal and physical abuse on sleep quality among individuals with SMDs. While both forms of abuse are harmful, only verbal abuse was significantly associated with poorer sleep. This suggests that emotional stress from verbal abuse may have a more persistent impact than the acute effects of physical harm. Previous studies have linked emotional distress to sleep disturbances [53-59], but our findings suggest that the type of bullying matters—offering a more nuanced understanding of how psychosocial stressors affect sleep in this population considered vulnerable. This distinction has not been well explored in prior research and could inform more targeted interventions.

This study provides new evidence that bullying by caregivers has a stronger association with sleep disturbances in individuals with SMDs than bullying by others. While previous research has documented the negative associations of interpersonal violence, few studies have differentiated the sources of abuse. Our findings suggest that the violation of trust within close caregiver relationships may be linked to more severe psychological distress, such as anxiety and depression, which in turn worsens sleep quality [60,61]. By contrast, bullying by outsiders, although harmful, may be buffered by support from trusted caregivers. This distinction highlights the unique vulnerability of patients with SMDs within dependent caregiving relationships and calls for greater attention to caregiver dynamics in both research and intervention design [62]. In the Chinese cultural context, family members often serve as both primary caregivers and key decision-makers in the care of individuals with mental disorders. This strong familial involvement, while supportive in many cases, may also amplify the psychological impact when abuse occurs within the family unit, due to the heightened expectations of filial piety and loyalty.

Family functioning serves as a positive factor against physical abuse for patients with SMDs. Effective family functioning is characterized by good communication, mutual understanding among family members, and the collective ability to cope with problems [63]. When family members have a better understanding of mental disorders, they are more likely to respond with patience and empathy, minimizing conflicts and misunderstandings [64-66]. This study adds to the literature by providing empirical evidence from a Chinese community-based sample, showing that better family functioning is significantly associated with lower physical abuse among individuals with SMDs. In China, where families are often the primary caregivers due to limited institutional resources, the quality of family dynamics plays a particularly critical role in the daily care and protection of individuals with mental illness. Our findings suggest that culturally tailored interventions focusing on improving family communication and mental health literacy could be especially effective in reducing violence and improving patient outcomes in the Chinese context.

This study found that middle-aged individuals (aged 45-64 y), only children, those with higher education, and patients with schizophrenia were more likely to experience bullying. The prevalence of mental disorders is higher among individuals aged 45 to 64 years, a group that often faces increased social isolation due to life transitions such as changes in employment or retirement [67,68]. Surprisingly, experiencing multiple types of bullying was found to be a positive factor, possibly because it enhances defense mechanisms and resilience. Studies also show that individuals with middle school education or above and only children are more likely to face bullying. Despite their educational background, patients with SMDs often struggle with social adjustment [69], and only children may seem more isolated due to lack of peer support [70]. Patients with schizophrenia are especially vulnerable, likely because their symptoms are more pronounced and noticeable in social settings [71]. These results emphasize the need for culturally sensitive, community-based strategies to protect groups considered vulnerable and improve mental health outcomes.

### Limitations

This study has several limitations, First, the small sample size and the fact that the sample was drawn from a specific community-based mental health management system in Shandong province may affect the stability and reliability of the results, limiting their generalizability to larger populations or to all individuals with SMDs in China. Moreover, the small subgroup sizes limited reliable stratified analyses, possibly missing spatial or socioeconomic differences. Second, the use of cross-sectional data prevents any causal inferences between variables. Third, data were collected through self-report and caregiver-assisted interviews, which may have introduced information bias, especially considering that caregivers could be sources of bullying themselves. Finally, due to data limitations, not all confounders could be included, leaving the possibility of residual confounding.

### Conclusions

This study highlights that self-stigma is associated with experiencing bullying among people with SMDs, while positive family functioning acts as a protective buffer. Notably, our findings add new understanding to the literature by identifying the differing associations of different bullying types on sleep quality—verbal abuse was linked to poorer sleep, whereas physical abuse showed no such long-term effect. Moreover, we found that bullying by caregivers was more strongly associated with negative sleep outcomes than bullying by others, emphasizing the crucial role of caregiving relationships. These results contribute novel insights, particularly within the Chinese context where individuals with SMDs often live with family members and rely heavily on them for daily care. Interventions in China must therefore prioritize self-stigma reduction and strengthen family-based support to improve the mental health and well-being of this underserved population.

## Abbreviations

GEE: generalized estimating equation
OR: odds ratio
SMD: serious mental disorder
SSCI-8: Stigma Scale for Chronic Illnesses, 8-item version

## Data Availability

The datasets generated or analyzed during this study are not publicly available due to being proprietary to the study team and not derived from a public database, but are available from the corresponding author on reasonable request.
